# Ontology-oriented retrieval of putative microRNAs in *Vitis vinifera *via GrapeMiRNA: a web database of *de novo *predicted grape microRNAs

**DOI:** 10.1186/1471-2229-9-82

**Published:** 2009-06-29

**Authors:** Barbara Lazzari, Andrea Caprera, Alessandro Cestaro, Ivan Merelli, Marcello Del Corvo, Paolo Fontana, Luciano Milanesi, Riccardo Velasco, Alessandra Stella

**Affiliations:** 1 Technology Park Lodi, Località Cascina Codazza, Via Einstein, 26900 Lodi, Italy; 2 IASMA Research Center, Via E. Mach 1, 38010 San Michele all'Adige (TN), Italy; 3 Institute for Biomedical Technologies (CNR), via Fratelli Cervi 93, 20090 Segrate (MI), Italy; 4 Institute of Agricultural Biology and Biotechnology (CNR), via Bassini 15, 20133 Milan, Italy

## Abstract

**Background:**

Two complete genome sequences are available for *Vitis vinifera *Pinot noir. Based on the sequence and gene predictions produced by the IASMA, we performed an *in silico *detection of putative microRNA genes and of their targets, and collected the most reliable microRNA predictions in a web database. The application is available at .

**Description:**

The program FindMiRNA was used to detect putative microRNA genes in the grape genome. A very high number of predictions was retrieved, calling for validation. Nine parameters were calculated and, based on the grape microRNAs dataset available at miRBase, thresholds were defined and applied to FindMiRNA predictions having targets in gene exons. In the resulting subset, predictions were ranked according to precursor positions and sequence similarity, and to target identity. To further validate FindMiRNA predictions, comparisons to the Arabidopsis genome, to the grape Genoscope genome, and to the grape EST collection were performed. Results were stored in a MySQL database and a web interface was prepared to query the database and retrieve predictions of interest.

**Conclusion:**

The GrapeMiRNA database encompasses 5,778 microRNA predictions spanning the whole grape genome. Predictions are integrated with information that can be of use in selection procedures. Tools added in the web interface also allow to inspect predictions according to gene ontology classes and metabolic pathways of targets. The GrapeMiRNA database can be of help in selecting candidate microRNA genes to be validated.

## Background

In plants, microRNAs (miRNAs) act as key regulators of several developmental pathways as well as of other molecular mechanisms, such as response to stress, or to environmental changes [1,2]. Plant miRNAs bind preferentially RNA transcripts of transcription factors, usually inducing their degradation. The events that lead to miRNA biogenesis are not completely elucidated, but critical steps are known, such as transcription by RNA polymerase II (POL-II) that produces primary miRNA transcripts (pri-miRs), cleavage of the pri-miRs to produce precursors (pre-miRs), and cleavage of precursors to obtain the miRNA:miRNA* duplexes. The two cleavage steps in animals are performed by the Drosha and Dicer enzymes. In plants no Drosha homologue has been detected, while homologues to Dicer were found in the nucleus as well as in the cytoplasm, suggesting that Dicer-like enzymes are involved in both cleavage steps [3]. Pre-miR stem-loop structures can be considered the hallmark of miRNAs and, because of this, methods for *in silico *detection of microRNAs in plant genomes are mainly based on their identification. Unfortunately, plant miRNA hairpins share their features with other classes of non-coding RNAs, like siRNAs, as well as with pseudo-hairpins that are present in the genome, particularly in repeat-rich regions. In animals, miRNA hairpins are shorter than in plants, being characterized by quite long loops and short stems. This helps discriminating between miRNAs and other hairpin-forming non-coding RNAs. Plant miRNA hairpins have an extremely variable length, spanning from about 60 to 500 bps, with an average of 160 nucleotides, and contain short loops and long stems. Furthermore, they do not exhibit preference with respect to the bulges position in the pre-miR structure [4]. This situation complicates the task of distinguishing pre-miRs from the other hairpin-forming non-coding RNAs, and leads to a very high proportion of false positives. Therefore, additional features distinctive of miRNAs must be considered. Conservation of mature miRNA sequences across species is a valuable source of validation. Although plant hairpin sequences are known to generally exhibit very low levels of sequence conservation (because the structure is usually more relevant than the nucleotide sequence), mature miRNA sequences are highly conserved even in phylogenetically distant species [5]. Nonetheless, conservation across species does not allow to identify species-specific miRNAs, thus, other features have also to be considered to discriminate among *in silico *predictions.

In grape, a set of 140 miRNAs has been inferred by similarity to already known plant miRNAs, and positioned on the Pinot noir genome sequence that was produced by the Genoscope Consortium [6]. In this paper we present the results of a *de novo *identification of miRNA genes and targets in the IASMA Pinot noir genome [7] that, with respect to the Genoscope genome, presents a much greater level of heterozygosity. Results from our analyses are stored in the GrapeMiRNA web database.

## Construction and content

### MicroRNAs *in silico *detection and *de novo *predictions selection

The second assembly of the high quality draft genome sequence of a cultivated clone of *Vvi *Pinot Noir that was produced at the IASMA [7] was used as reference sequence. Gene positions on the genome, as well as intron/exon boundaries and information concerning repeats and other features were based on gene predictions that were carried out at the IASMA. The FindMiRNA algorithm [8] was employed to scan the grape genome for the presence of putative miRNA::target couples. FindMiRNA identifies putative miRNA genes in intergenic regions, with targets in gene sequences. In our analysis, putative miRNA genes were searched on both strands in the intergenic regions, while putative miRNA targets were searched within the gene sequences, encompassing 300 bp of both upstream and downstream boundaries. Repeats, tRNAs and low quality regions were masked prior to the analysis.

The FindMiRNA analysis produced 785,441 microRNA predictions. These were parsed and used to populate a MySQL database. As expected, the number of predictions obtained with FindMiRNA greatly exceeded the expected ratio of miRNAs in the grape genome, necessitating the application of a selection procedure to reject the less reliable hits. A first filtering step was performed applying low stringency filters to four parameters. We selected ≤ -28 kcal/mol as the lowest stability limit for the predicted miRNA-target pair as estimated by FindMiRNA from the minimum free energy (MFE) of the miRNA::target duplex, and ≥ 45 bps as the limit for precursor length. Only miRNAs with percentages of G+C content between 33 and 65 were considered. Furthermore, based on the assumption that plant miRNAs are likely to have an uracil residue at the 5' end of their mature sequence [9], only the predictions having an uracil at the 5' end or in its boundaries (bases -2, -1, 0, +1 and +2 with respect to the predicted mature miRNA 5' end) were retained in the filtered database. The tolerance in uracil position was adopted to overcome the inability of FindMiRNA to precisely assign the position of the miRNA 5' end. After this selection step, the resulting subset contained 227,369 predictions (less than 30% of the total predictions), and was used to populate the 'mirna' database table. Classification of predictions with respect to the target position (in exons, introns, or in 5' or 3' UTRs) was performed, and predictions in the mirna table were flagged accordingly. 5' or 3' position of the predicted mature miRNAs on precursor sequences as well as the precursor strand carrying the mature miRNA were also inferred and added to the database. To further investigate FindMiRNA predictions we proceeded with two additional parallel analyses, the former based on comparative genomics (see later), and the latter on distinctive sequence and structural features of the hairpins. The experience of Kwang Loong and Mishra [10,11] in identifying features crucial for miRNA distinction allowed us to apply to our predictions five parameters having precise confidence intervals both in vertebrates and plants. Among the precursor features of Kwang Loong and Mishra, we selected length, G+C percentage, MFE of the hairpin secondary structure normalized according to the precursor length (MFEs), MFEs/G+C content percentage (MFEI), and base-pairing propensity (P(S)): i.e. the percentage of nucleotides forming complementary base pairings within the hairpin structures. Considering that in plants miRNAs mostly target gene exons, we focussed our attention on the 54,143 predictions having targets in exons (referred to as 'exon predictions', and stored in the 'mirna_exon' database table), and calculated values for these parameters to be added to the database. Self containment scores were also calculated with the Selfcontain algorithm [12]. The property of self containment can be defined as the tendency for an RNA sequence to maintain the same optimal secondary structure regardless of whether it exists in isolation or is a substring of a longer sequence of arbitrary nucleotide content. MiRNAs are known to have very high self-containment scores (an average of 0.9, the score ranging from 0 to 1) when compared to other functional RNAs.

To define grape-specific confidence intervals for all the parameters calculated on FindMiRNA exon predictions, we downloaded the complete *Vvi *miRNA dataset available at miRBase version 12.0 [13] (based on the Vvi Genoscope genome), to be used as the reference dataset for thresholds setting. The 140 *Vvi *miRNAs were inspected according to the seven parameters chosen for prediction selection, and thresholds were set for each parameter as to retain most of the miRBase miRNAs (Table 1). Applying these cutoffs to FindMiRNA exon predictions, 5,778 predictions were selected (less than 13% of the total exon predictions) and included in the 'selected predictions' dataset. As miRNA detection was carried out on both strands of the genome, FindMiRNA selected predictions encompassed 2,500 and 3,278 miRNA genes on the grape forward and reverse genome strands, respectively. In several instances, the hairpin structure was present on both strands in the same region, resulting in multiple predictions for the same genome position. Unfortunately, positions that refer to the same genome region in forward and reverse orientation are not easily recognizable in FindMiRNA outputs, as reversed-complementary genomic contigs are re-numbered in 5'-3' direction. As a consequence, it can be assumed that the overall number of genome positions where predictions of miRNA genes were recovered is less than 5,778.

**Table 1:** Parameters calculated on FindMiRNA predictions and thresholds adopted for selection of predictions

**Parameter name**	**Parameter description**	**Parameter cutoff**	
		**mirna_exon**	**selected_predictions**

Position in precursor	Indicates the miRNA* position (at the precursor 5' or 3' end)		

Strand	Indicates the precursor strand where the mirna* is located (+ or -)		

5'U present	Retains only those records for which a U residue is present in the -2, -1, +1 and +2 positions with respect to the 5' nucleotide of the predicted miRNA sequence.	yes	yes

miRNA % G+C content	G+C percentage in the mature miRNA sequence	≥ 33 and ≤ 65	≥ 33 and ≤ 65

Precursor length	Length of the precursor in base pairs	≥ 45 bp	≥ 72 bp and ≤ 442 bp

MFE	Minimum free energy: estimated stability of the miRNA-candidate::target duplex	≤ -28	≤ -28

Precursor % G+C content	G+C percentage in the precursor sequence		≥ 35 and ≤ 66

Precursor homology %	Percentage of homology in the precursor hairpin		> 50

Length normalized MFE (MFEs)	Minimum free energy of the precursor secondary structure normalized according to precursor length		≤ -0.23 and ≥ -0.66

MFEI	MFEs/% G+C content		≤ -0.005 and ≥ -0.012

Self containment	Precursor self containment index, as calculated by Selfcontain		≥ 0.89

A list of the parameters that were calculated for FindMiRNA predictions. Cutoffs that were adopted to select predictions that are stored in the mirna_exon and selected_predictions tables are indicated in the rightmost columns.

### Comparing predictions to the Arabidopsis and grape Genoscope genomes

The PrecExtract program [8] allows to scan other genomes with FindMiRNA predictions. PrecExtract doesn't take into account putative miRNA::target pairings, but it detects mature miRNA sequences proposed by FindMiRNA that fall in a genome region hosting a hairpin structure that satisfies a maximum energy threshold and has at least 70% of the mature miRNA and its complement binding. We used PrecExtract to compare the 5,778 selected predictions to the *Arabidopsis thaliana *(*At*) and to the grape Genoscope genomes as downloaded from the TAIR [14] and Genoscope [15] web sites, respectively. Searching for full-length identities between predicted miRNAs and the other genomes, only a limited number of hits was retrieved. Conversely, when PrecExtract considered core sequences of predicted miRNAs where two bases both at the 5' and 3' end were removed, a more consistent number of hits was obtained (354 and 691 for *At *and grape Genoscope, respectively), several with more than one match with the compared genomes. The dramatically higher number of hits retrieved using miRNA core sequences can be explained considering that FindMiRNA assigns with a low degree of precision the miRNA 5' end, as clearly stated by FindMiRNA authors. Based on this, we preferred to run PrecExtract on miRNA core sequences rather than allowing mismatches all along the miRNA sequence.

In parallel to the PrecExtract analysis, comparison of predicted mature miRNAs to the *At *and Genoscope genomes was also carried out with BLAST [16]. Only full-length BLAST similarities with fewer than three mismatches in the 5' and/or 3' ends and no gaps were taken into account, and 218 and 173 hits were retrieved for the *At *and Genoscope genomes, respectively. MiRNAs retrieved both by the PrecExtract and BLAST analyses were 81 for the *At *genome and 106 for the Genoscope genome, and only 28 showed matches with both methods on both genomes (IDs: 47802, 47806, 91434, 129414, 144854, 184639, 215697, 217048, 229160, 233378, 272542, 275873, 313024, 327361, 332125, 398759, 502648, 552546, 579252, 590679, 590939, 631942, 644118, 653750, 665068, 702837, 715369, 733207). From the biological point of view, the two analyses are not equivalent. BLAST analysis highlights matches with not more than three external mismatches on the full miRNA sequence, regardless of the presence of a hairpin in the region. PrecExtract takes into account miRNA-like secondary structures but with our low stringency settings allows up to four terminal mismatches (two at each end). Merging the two analyses, hits that fall in putative hairpins and having not more than three terminal mismatches are retrieved. These miRNAs can be considered good candidates for validation.

### Comparing predicted precursors to grape EST sequences

In plants, pri-miRs are produced by POL-II and are capped and polyadenylated [17]. Pri-miRs are processed and converted to pre-miRs, that are subsequently cleaved to generate miRNA:miRNA* duplexes. Being polyadenylated, primary miRNA transcripts should be recoverable in EST collections. Even if previous studies suggest that miRNAs should constitute nearly 1% of predicted protein-coding genes [18], their representation in EST datasets is usually much lower, being under 0.01% [5].

The current explanation is that the procedures that are carried out during EST libraries preparation contribute to lower the amount of cloned miRNA precursors. Furthermore, the possible rapid processing of pri-miRs in the cell may also contribute to the decreased representation of their transcripts in cDNA libraries. Translation of pri-miRs leads to short peptides that cannot be annotated against conventional protein databases. Even considering the over-mentioned problems, identification of miRNA precursors in ESTs is a tool which can improve knowledge of miRNA biogenesis. In Arabidopsis, evidence of the presence of more than one miRNA within a single transcript has been provided by Zhang *et al *[5], suggesting that also in plants clustered miRNAs can be transcribed as polycistrons, as already observed in animals [19-22].

At the DFCI grape gene index (VvGI) [23], 78,976 unique sequences that encompass 347,879 EST and 25,497 ET sequences are available. This collection represents a comprehensive overview of the grape transcriptome, and it thus merits scanning for the presence of miRNA precursors. We compared FindMiRNA putative selected precursors to the VvGI dataset by BLASTn, and recovered 152 ESTs perfectly matching 359 predicted precursors, reflecting both the redundancy that is intrinsic to the FindMiRNA output, as well as the possibility to recover the same precursor in more than one genome position. We annotated the matching ESTs and retrieved eight ESTs without similarity to the NCBI nr protein database, suggesting that predictions that match these ESTs are good candidates for validation (Table 2). Of the 32 precursors matching the un-annotated ESTs, two were flagged as miR-172, one as miR-159 and one as miR-397 (see later). In most cases, more than one precursor matching the same EST in almost fully overlapping regions was recovered, due most probably to the abundance of predictions proposed by FindMiRNA. No transcripts containing more than one miRNA or more copies of the same miRNA were detected.

**Table 2:** MicroRNA predictions matching un-annotated ESTs

**VvGI EST Identifier**	**EST sequence length**	**miRNA ID**	**Precursor length**	**Precursor position in EST sequence**	**Orientation**	**miRNA**
EC979165 (singlet)	296	279806	108	144–251	+/+	miR-397
		315332	104	249–146	+/-	
FC057876 (singlet)	429	193758	100	190–289	+/+	
		272427	100	190–289	+/+	
		383142	100	190–289	+/+	
		389989	100	190–289	+/+	
TC83445 (contig)	755	746258	116	80–195	+/+	
TC84091 (contig)	657	4592	122	362–483	+/+	miR-172
		256855	122	362–483	+/+	miR-172
		594486	112	478–367	+/-	
TC86536 (contig)	1066	126771	83	566–484	+/-	miR-159
		567065	85	567–483	+/-	
TC89289 (contig)	560	191738	124	293–170	+/-	
		191739	124	293–170	+/-	
		205575	124	293–170	+/-	
		229080	124	170–293	+/+	
		234968	132	166–297	+/+	
		234999	132	166–297	+/+	
		238365	124	170–293	+/+	
		238366	124	170–293	+/+	
		247518	132	166–297	+/+	
		247519	132	166–297	+/+	
		247538	132	166–297	+/+	
		247539	132	166–297	+/+	
		553182	124	293–170	+/-	
		553183	124	293–170	+/-	
		562443	124	293–170	+/-	
		750086	126	294–169	+/-	
		750087	126	294–169	+/-	
		761906	126	294–169	+/-	
TC90232 (contig)	724	635488	137	75–211	+/+	
TC96134 (contig)	425	256915	140	153–14	+/-	

Predictions matching the VvGI un-annotated ESTs. VvGI identifiers are given in the leftmost column, together with the classification as singlet or contig, as from the VvGI dataset. In the rightmost column, predictions matches to known miRNAs are displayed.

Predictions matching ESTs corresponding to known proteins need to be checked with caution. The consideration of a sample subset, in fact, indicated that these predictions are likely to reflect problems in gene assignments. For instance, the 89 predictions ranked in Contig6 according to precursor similarity should be discarded, because their putative precursors are part of a gene sequence not recognized by gene predictors because the start of the contig lies within the gene coding sequence. When compared to the NCBI protein nr database, both the homologous EST and the genomic region encompassing the putative precursors showed a significant homology with the *Populus trichocarpa *CCHC-type integrase: a zinc finger, retroviral-type protein. As multiple copies of this gene or its paralogs can be retrieved in the genome, multiple putative targets were spotted by FindMiRNA, and a high number of false predictions were generated. Predictions matching to annotated ESTs were not removed from the database, but were flagged with the EST name.

### Positioning of known miRNAs on the grape genome

Four BLAST analyses were carried out to compare FindMiRNA predictions to known miRNAs that are collected in miRBase: mature miRBase sequences were blasted versus FindMiRNA mature sequences, target sequences, and precursor sequences, and miRBase precursor sequences were blasted versus the IASMA Pinot noir genome. Following this last comparison, positions of precursors on the genome were retrieved and compared to positions of precursors identified by FindMiRNA, and predictions having mature sequence boundaries internal to the miRBase precursor genomic position were flagged in the database. Three out of the four BLAST analyses were performed using the Vvi miRBase dataset, while BLAST versus FindMiRNA precursor sequences was carried out using the whole miRBase mature sequence dataset, completed with the new Arabidopsis miRNAs proposed by Rajagopalan *et al *[9]. In spite of this, no significant matches to additional miRNA families, apart from those present in the *Vvi *dataset, were retrieved. In all BLAST analyses only full length homologies with no gaps and not more than three mismatches were retained. On the whole, 65 predictions showing similarity with *Vvi *miRBase entries were retrieved, encompassing 17 out the 28 miRNA families that are represented in *Vvi *miRNAs (Table 3).

**Table 3:** FindMiRNA predictions matching known microRNAs

**Prediction ID**	**Vvi-miRBase mature vs FindMiRNA mature**	**Vvi-miRBase mature vs FindMiRNA targets**	**Vvi-miRBase precursors vs IASMA genome**	**all miRBase mature vs FindMiRNA precursors**
464982	Vvi-miR156	Vvi-miR156	Vvi-miR156	miR156
116355	Vvi-miR159	Vvi-miR159		miR159
116356		Vvi-miR159	Vvi-miR159	miR159
126771		Vvi-miR159		miR319
29160	Vvi-miR160	Vvi-miR160	Vvi-miR160	miR160
304077				
486346				
43452		Vvi-miR160		miR160
317194				
496248				
496249				
25252			Vvi-miR164	
680841				
37580		Vvi-miR164		miR164
399187	Vvi-miR171	Vvi-miR171		miR171
412275		Vvi-miR171		miR171
412283				
412284				
412286				
412287				
368753			Vvi-miR171	miR171
399184				
378732		Vvi-miR171		miR171
4592	Vvi-miR172		Vvi-miR172	miR172
729515				
729516				
256855	Vvi-miR172	Vvi-miR172	Vvi-miR172	miR172
256857				
256858				
256859				
256856	Vvi-miR172	Vvi-miR172		miR172
729517	Vvi-miR172			miR172
567062		Vvi-miR319		miR319
567063				
567065				
21821	Vvi-miR393	Vvi-miR393	Vvi-miR393	miR393
534183				
749266			Vvi-miR395	
749267				
749268	Vvi-miR395		Vvi-miR395	miR395
749269				
760872				miR395
760873				
760874				
760875				
51691	Vvi-miR396			
353241	Vvi-miR396			miR396
279806	Vvi-miR397	Vvi-miR397	Vvi-miR397	miR397
315332				miR397
575210	Vvi-miR399		Vvi-miR399	miR399
575211				
584266				miR399
157143				miR403
290554		Vvi-miR403		miR403
290555				
765421		Vvi-miR414		
93427			Vvi-miR477	
274857				
752076				
752079				
752084				
560409			Vvi-miR479	
50626			Vvi-miR535	
220937		Vvi-miR828		miR828
628384	Vvi-miR828		Vvi-miR828	miR828

Predictions matches to known miRNAs according to the four adopted procedures. Datasets used for BLAST comparisons are given in column headers.

Comparison between FindMiRNA and miRBase precursors sharing an overlapping genome position revealed differences in sequence length. By a large majority, miRBase sequences are longer. The difference is in part explained considering that miRBase stem-loop sequences include the pre-miR and some flanking sequence of the presumed primary transcript, whereas FindMiRNA predictions describe only the putative pre-miR sequences. In this case, similarity in our predictions both at the precursor and at the mature miRNA level were found. In other instances, similarity was evident only at the precursor level. This was the case when putative mature sequences different from those collected in miRBase were proposed by FindMiRNA in regions suitable to form more than one hairpin structure. A third situation corresponds to similarities encountered only across mature sequences. This could be explained by the fact that two different genomes were considered, with the IASMA one having a much greater level of heterozygosity, where differences in precursor sequences can exist as alternative haplotypes.

Comparing all miRBase mature sequences to FindMiRNA precursors with our thresholds (not more than three mismatches and no gaps with the full-length mature sequence) matches to all the 28 represented miRNA families were originally retrieved, involving 121 predictions. Hits to 12 families were discarded following our further analysis, where only matches with positions not more than three bps distant from the precursor 5' or 3' end were retained (table 3). When the discarded dataset – encompassing predictions with hits to miRBase mature sequences internal to the core of the precursor sequence – was analyzed according to more stringent criteria, and only full-length perfect matches were considered, matches to three miRNA families (miR151, miR153 and miR170) were lost, while matches to eight other families, apart from those presented in Table 3, were still recovered. It is worth noting that four of these families (miR132, miR136, miR140 and miR157) are not included in miRBase for *Vvi*. A possible explanation for this situation is that the involved predictions fall in genomic regions that are prone to form hairpin structures, and FindMiRNA failed to recover the ones leading to the matching mature sequences. Reasons for this failure could be for example ascribed to missing corresponding target sequences.

To further investigate the prediction accuracy of FindMiRNA combined with the chosen selection parameters and thresholds, covariance models from 46 known microRNA families were deduced from RFam 8.1 [24] and used to search the grape genome for homologues to known structural RNA families with the Infernal software package (data not shown) [25]. Infernal results were compared to FindMiRNA predictions according to the genome coordinates, but even if many of the similarities identified by BLAST were confirmed, no additional significant hit was retrieved.

### Analysis of genes involved in microRNA biogenesis

In the grape IASMA genome, 56 genes showing homology with Arabidopsis Dicer-like proteins (DCL1, DCL2, DCL3 and DCL4), Argonaute (AGO1, AGO2, AGO4, AGO6 and AGO7), Hyponastic Leaves 1 (HYL1), Nuclear RNA Polymerase D (NRPD1a and NRPD2a), RNA-dependent RNA Polymerase (RDR2 and RDR6), Zwille (ZLL), and PAZ domain-containing protein/piwi domain-containing protein were identified by BLASTp (E-value < e^-11^) [3]. In plants, messages for Argonaute and other biogenetic and effector proteins (i.e. DCL1) are considered as conserved miRNA targets, together with messages for a variety of transcription and stress response factors [9]. The selected predictions dataset was scanned for the presence of putative miRNAs targeting the 56 over-mentioned genes, and five predictions were retrieved (IDs: 42291, 238196, 385559, 474626, and 761661), all targeting genes belonging to the Argonaute family, and none matching known miRNAs. The 42291 and 761661 predictions refer to the same putative miRNA, targeting two different Argonaute genes carrying identical target sites. An Arabidopsis homolog to this miRNA was retrieved both by PrecExtract and by BLAST. An Arabidopsis homolog was identified by PrecExtract also for prediction 385559, that in addition to targeting the AGO1 gene also targets a second gene coding for a Pentatricopeptide (PPR) repeat-containing protein.

In recent studies, Rajagopalan *et al*. [9] provided evidence of the presence of a miRNA gene (miR838) overlapping DCL1 intron 14. Thus, we decided to perform a FindMiRNA run to detect eventual putative miRNAs in the introns of the 56 genes involved in miRNA biogenesis, with targets in grape gene exons. The same thresholds that were used to prepare the selected_predictions dataset were applied to the FindMiRNA output, and 99 predictions – giving rise to 17 precursors similarity groups – were retrieved and stored in the selected_intron_predictions table. Among these, no prediction matching either the new miRNAs described by Rajagopalan *et al*. or the miRBase dataset was recovered. Intron predictions are available at the GrapeMiRNA web site.

### Predictions ranking

In order to investigate the prediction dataset with respect to the distribution of miRNA genes in the genome and to recognition of target genes, ranking of predictions was necessary. Predictions were grouped according to target identity, precursor position in the genome, and precursor sequence similarity, and results were stored in the database. Ranking according to target identity allows identifying different miRNAs that bind identical targets, as well as different grape genes that share common miRNA targets and genes with multiple copies of the same target. Identical target ranking produced 864 groups encompassing 3,026 out of the total 5,778 predictions, the other 2,752 remaining ungrouped. Thus, the selected predictions encompass 3,616 different putative targets (864 + 2,752). The second procedure, that was carried out with an in-house developed script, aimed at the identification of precursors with start positions within 3 bp in the genome. 780 groups encompassing 2,228 predictions were obtained, while 3,550 precursors remained ungrouped. This means that according to their position in the grape genome, the selected predictions can be ranked in 4,330 groups (780 + 3,550). Predictions ranking according to precursor similarity was performed with CAP3 (Parameters: -p 98 -o 25) [26]. This procedure identifies miRNAs that are present in more than one genome position. Of course, multiple predictions generated by FindMiRNA for regions where more hairpin structures are putatively present fall in the same precursor similarity group, but should be considered alternative structures of the same putative miRNA and not multiple independent miRNAs. Ranking predictions according to precursor similarity resulted in 857 groups encompassing 4,060 predictions (2,233 of which also belonging to position groups): in total, 2,575 similarity groups were obtained (857 groups + 1,718 ungrouped precursors). Combining results from the three procedures, an exhaustive view of miRNA genes and targets distribution across the genome was obtained. It is worth noting that precursor predictions that fall in the same genome region but on opposite strands cannot be grouped with the position ranking tool, but fall into the same precursor similarity group.

As an example, we report here the analysis of one of the most numerous groups obtained by similarity ranking of precursor sequences (precursor_Contig207). This similarity group contains 73 miRNA predictions targeting 32 genes, with 24 different putative targets (i.e. it encompasses targets from 24 target ranking groups). The overall predictions are ranked in 16 precursor position groups. Some of these groups have consecutive numbers, indicating that they fall in genomic regions where multiple consecutive hairpin structures are present, all passing the selected parameter cutoffs, with very close start positions but spanning a region wider than three base pairs. These are proposed by FindMiRNA as possible miRNA genes. If consecutive position groups are further ranked, and corresponding predictions on reversed genomic contigs are also merged, seven groups are obtained, which can be assumed to correspond to seven similar miRNA genes present in different genomic regions. 25 out of the 32 target genes associated to precursor_Contig207 are annotated as putative non-LTR retroelement reverse transcriptases, one as an ankyrin-repeat containing protein and one as DNA-directed RNA polymerase, while the 5 remaining genes do not have a significant annotation. Due to the redundancy of predictions, target genes are targeted by one to seven putative miRNA genes, but they mainly contain single targets, or two tandem targets separated by about 100 base pairs.

An example of identical target grouping is CL863. This group includes 56 predictions referring to a couple of genes (fgenesh.VV78X016421.10_1 and fgenesh.VV78X210321.6_1), both annotated as receptor protein kinase-like proteins. The two genes bear the same target in similar positions (from bp 3383 to 3401 for the former, and from bp 3377 to 3395 for the latter) and are putatively targeted by 28 miRNA genes that are interspersed all along the genome. None of these miRNA genes seems to be repeated in tandem, as only one genomic contig includes two miRNA copies, and these are very distant one from the other. All putative mature miRNAs are on the forward strand of the respective gene, at the 5' end.

### Structuring the GrapeMiRNA web database: the text search interface

Considering the large amount of data stored in the GrapeMiRNA database, a web interface was prepared to provide free access to all information. Our intention was to produce a web site with tools and facilities to allow users to retrieve information according to multiple criteria. With this aim, we focussed on two main aspects: retrieval of predictions according to their features and parameter values, and retrieval of predictions according to biologically relevant features of the targeted genes. Even if the GrapeMiRNA database contains all the predictions that were produced by FindMiRNA, the online version is limited to the 5,778 selected exon predictions that are supposed to represent the most reliable subset of the total FindMiRNA output (Table 4). At the GrapeMiRNA web site a text search page is available where users can perform queries on a number of fields. Queries can be restricted to subsets of predictions (i.e. predictions with homologues in the *At *or Genoscope genomes, or matching already known *Vvi *miRBase miRNAs), or to selected ranking groups. In query outputs a table is displayed including the most relevant information for each prediction matching the query terms. PrecExtract results are included in the output, when present, as well as the number of matches retrieved by BLAST in comparisons between FindMiRNA mature miRNAs and the *At *and the Genoscope genomes.

**Table 4:** The selected predictions dataset

Total number of predictions	5,778
Position assembled precursors	4,330
Homology assembled precursors	2,575
Target ranking groups	3,616
Position in precursor	5'end: 2,926 3'end: 2,852
Strand	+ strand: 2,642 - strand: 3,136
PrecExtract vs Arabidopsis genome	354
Mature miRNA homologues to Arabidopsis genome (BLAST analysis)	218
PrecExtract vs Genoscope grape genome	691
Mature miRNA homologues to Genoscope grape genome (BLAST analysis)	173
BLAST homologues to grape ESTs	359

Composition of the dataset included in the selected_predictions table. Selected predictions are available at the GrapeMiRNA web site.

**Figure 1:**
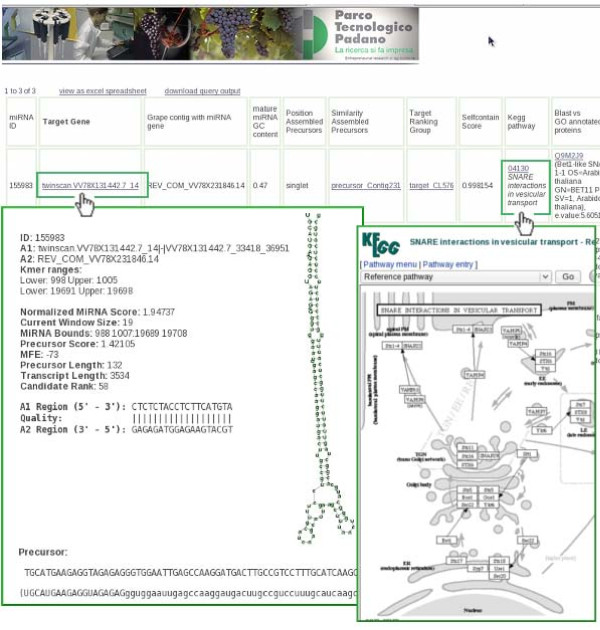
**The GrapeMiRNA web interface**. An example of output display at the GrapeMiRNA web database.

Predictions matching EST sequences are flagged with the name of the corresponding sequence, and matches to *Vvi *miRNAs included in miRBase are also given. In the output table, miRNA predictions matching the query terms are displayed. It is worth noting that predictions having more than one hit to other genomes by PrecExtract are proposed in multiple lines. Thus, the number of retrieved hits can be larger than the number of corresponding predictions. In the output, links to other web pages are provided, where particular aspects are deepened. For instance, clicking on the target gene name of each prediction leads to a page where the FindMiRNA output is displayed, together with the miRNA, miRNA* and precursor sequences, and the hairpin secondary structure, produced on the fly by RNAFold [27] (Figure 1). Conversely, a click on the links that are given in the 'Position assembled precursors', 'Similarity assembled precursors' and 'Target ranking group' columns leads to tables containing all the predictions matching the selected ranking group. Furthermore, in the 'Similarity assembled precursors' pages, precursor sequences are displayed in multifasta format, and CAP3 [26] (parameters: -p 96) is run on the fly on the similarity-grouped precursors to display alignment results.

A group of options included in the text search page allows to select predictions according to the targeted gene features. In the 'text search' page, targeted genes can be retrieved according to their annotation, or to their best BLAST hit ID. Furthermore, the possibility to retrieve grape targeted genes belonging to metabolic pathways of interest is also implemented. Query outputs can be downloaded or directly visualized with ordinary spreadsheets.

At the text search page, an option is given to visualize the predictions contained in the selected_intron_predictions table (i.e. predictions in introns of genes involved in miRNA biogenesis), or the table can be downloaded in Excel-compliant format.

### Statistics on ontologies distribution

With the aim to allow investigating predictions according to the annotation, ontology class, or metabolic pathway of targets, a procedure was set to relate grape genes to corresponding UniProt [28], Gene Ontology (GO) [29,30], and KEGG pathways [31] identifiers (IDs).

The 33,514 genes predicted by the IASMA on the Pinot noir genome were annotated by BLASTx (e-value cutoff: e^-10^) versus a customized version of the UniProtKB database [28], where entries from genome sequencing projects having non-descriptive annotations and entries lacking cross-references to GO IDs were discarded. 26,962 significant hits were retrieved, representing the 80.45% of the total gene predictions. Based on GO IDs that are associated to UniProt IDs, significant best BLAST hits can be used to classify grape genes in ontology classes.

Based on data contained in the Gene Ontology Annotation (GOA) Database [32] and in the Gene Ontology Database [29], Perl scripts were prepared to create a local database with all the protein-GO associations including no-direct links due to "is_a" relations among different GO elements. Information contained in the database tables was used to produce statistics on the ontologies distribution. According to the distribution of GO IDs in the GO Direct Acyclic Graph (DAG), statistics were created representing the participation of the grape gene set in the different GO categories. As for the grape genes collection, GO statistics were also created for the putative target genes dataset that was deduced from FindMiRNA output. Each time a gene is targeted by a FindMiRNA prediction, it is included in this dataset, so that genes that are targeted more than once are represented in multiple copies. Comparing distributions of hits in GO classes from the two sets of statistics allows to highlight over- or under-targeted categories, and to retrieve corresponding predictions. In the GrapeMiRNA web tool, graphical display and browsing of ontology classes is obtained via the PHP-based web interface, that produces graphical bars and matching ontologies percentages upon users' requests. GO classes are represented as proportional bars, carrying aside the percentage of hits matching each class. Bars can be clicked to move hierarchically across categories, and hits matching each category can be retrieved. To facilitate picking up of GO categories where statistically significant differences between the two datasets are observed, a chi-squared analysis was performed on a generic GO slim [33], and a table of results is published at the corresponding web page. In the table, the number of hits for each dataset matching the GO categories contained in the GO slim, normalized to a sample dataset of 30,000 sequences, is displayed. In the table nodes, links to the corresponding proportional bars are active, so that retrieval of matching hits is granted.

UniProt identifiers associated to annotated grape sequences were used to relate sequences to the 345 molecular pathways that are described at the KEGG Pathway database [31]. UniProt-pathway inter-relationships were deduced from association files available at the KEGG ftp site.

## Utility and discussion

### *De novo *microRNA identification and predictions selection

The FindMiRNA algorithm was used to predict putative miRNAs and their targets in the grape genome. FindMiRNA identifies miRNA-like hairpin structures in intergenic regions having putative targets in genic sequences, and its output is thus independent from comparative genomics approaches. To assess the presence of predictions that are conserved across species, FindMiRNA's authors implemented the PrecExtract program, that looks for the presence of putative pre-miRs having the same mature sequences as those predicted by FindMiRNA in other genomes. Unfortunately, plant pre-miRs share their features with other non-coding RNAs and other types of foldback elements, so these structures are also reported in FindMiRNA output together with putative miRNAs, as no other miRNA-distinctive feature is taken into account during the FindMiRNA analysis. Furthermore, difficulties are encountered by the program in assigning the 5' end of the miRNA sequence based on miRNA-target base pairing. As a result of these considerations, the FindMiRNA output contains a plethora of predictions that need to be validated with complementary methods to select the most reliable miRNA-like structures. In recent years, knowledge about miRNA structure and features has been expanded, and new parameters can be adopted to discriminate among predictions. Applying combined filters to our predictions dataset, only about 0.74% of the original FindMiRNA predictions was selected. Among the parameters that were adopted to discriminate among predictions, the self-containment score was the most efficient in slashing the predictions number. The resulting dataset still contains a significant intrinsic redundancy, due to the fact that multiple predictions are produced in overlapping regions when more than one hairpin structure can be predicted, or when miRNA-like structures are detected on both genomic strands. According to these assumptions, predictions ranking was mandatory. The three ranking procedures that were applied to the dataset of selected predictions allow not only to focus on miRNA-containing genomic regions, but also to focus on genes sharing the same targets or on miRNA families.

### Predictions validation by comparison to other sequences

After selection of predictions according to their sequence and structural features, an effort was made to classify predictions and to validate FindMiRNA combined with the adopted parameters and thresholds as effective instruments for *de novo *identification. Comparisons of predictions with previously described miRNAs, EST sequences and other genomes were performed with the dual aim of *in silico *validation and individuation of good candidates for *in vitro *analysis. The selected predictions dataset contains members from only 17 out the 28 miRNA families that are reported for grape in miRBase. Even if all the grape miRBase mature miRNA sequences can be recovered by BLAST analysis on the IASMA Pinot noir genome (data not shown), FindMiRNA and further prediction selection with our thresholds failed to recover more than one third of the represented miRNA families. Nonetheless, it is worth noting that searches for homologues to known structural RNA families that were carried out with Infernal confirmed these results without identifying possible members from further miRNA families (data not shown). This problem can partially be ascribed to sequence or gene prediction differences between the two grape genomes, but it is likely that part of the missing families are not detected due to procedural problems. An insight in the 'mirna' database table reveals that no additional miRNA family is reported with respect to those included in the 'selected_predictions' table, thus failure in their detection cannot be ascribed to excessively stringent cutoffs.

Comparisons of FindMiRNA predictions with the *At *and grape Genoscope genomes by PrecExtract and BLAST were performed to validate predictions as cross-species conservation is an established trait for miRNAs. Nonetheless, the independency of the FindMiRNA algorithm from cross-species validation grants the possibility to recover species-specific miRNAs, that are not detected by comparative genomics-based approaches. 858 predictions having matches with almost one of the two compared genomes were individuated by PrecExtract, 189 of which were also confirmed by BLAST. By comparison of FindMiRNA output precursors with ESTs, 32 putative precursors matching eight un-annotated ESTs were identified. These are likely to correspond to miRNA genes which pri-miRs were cloned to ESTs.

## Conclusion

Even if the adopted procedure was not sensitive enough to recover all known miRNA families in the IASMA genome, among the selected predictions a significant number of homologues to the *At *and Genoscope Pinot noir genomes was recovered, and precursors sharing their sequence with putative priMIRs that were cloned to ESTs were also retrieved. According to these results, we can assume that it is likely that predictions available at the GrapeMiRNA web site contain a discrete number of good candidates for validation. Nonetheless, eventual *in vitro *validation studies must take into account the possibility that mature miRNA sequences proposed by FindMiRNA could be almost in part imprecise, and their boundaries should be carefully inspected or broadened.

The GrapeMiRNA public interface was structured to allow researchers to focus on genomic regions or targeted genes of interest, and retrieve corresponding miRNA predictions. Even if predictions require validation and must be inspected with caution, we believe that the computational effort that was required to perform the FindMiRNA analysis and the accessory analyses that were carried out to enrich predictions-related information cannot be easily reproduced without the aid of sophisticated hardware configurations. The overall procedure that led to the completion of the GrapeMiRNA web database involved the preparation of several parsers, scripts and accessory programs that allowed extracting relevant results from programs' outputs and organizing them in the database tables. The FindMiRNA, PrecExtract and Infernal analyses were carried out on a computer cluster, using a significant number of nodes. Further tools were developed to relate predictions to information concerning targeted genes, including the statistical analysis on distribution of grape genes and putative target genes in ontology classes. The resulting database includes a significant amount of data that can be of use in mining miRNA distribution across a plant genome and in selecting candidate miRNAs for *in vitro *validation.

## Availability and requirements

The GrapeMiRNA web database is freely available at .

## Authors' contributions

BL defined the miRNA detection, prediction selection and analysis procedures, performed data analyses, designed the web interface contents and drafted the manuscript; ACa structured the database and wrote all the accessory programs, implemented the GO statistics tool and the web interface; ACe contributed to the gene prediction and the functional annotation of *Vitis vinifera *genes and genome features, contributing to the preparation of the manuscript; IM enabled the parallel computation of the microRNA prediction software on the cluster infrastructure; MDC helped in the construction of the GrapeMiRNA web interface; PF was involved in gene assignment procedures; LM granted the access to the computational facilities and the maintenance of the bioinformatics resources; RV coordinated the *Vitis vinifera *genome sequencing activity, gene prediction and annotation and contributed to the critical reading of the manuscript; AS guided and coordinated the execution of the project and critically revised the manuscript. All authors read and approved the final manuscript.
